# Supplementary material for: Clinical Utility of SARS-CoV-2 Antibody Titer Multiplied by Binding Avidity of Receptor-Binding Domain (RBD) in Monitoring Protective Immunity and Clinical Severity
Source: Viruses. 2023 Jul 30;15(8):1662. doi: 10.3390/v15081662 (PMC10459795; doi:10.3390/v15081662)
Supplement: Supplementary file 1 [file viruses-15-01662-s001.zip › viruses-2497507-supplementary.pdf]

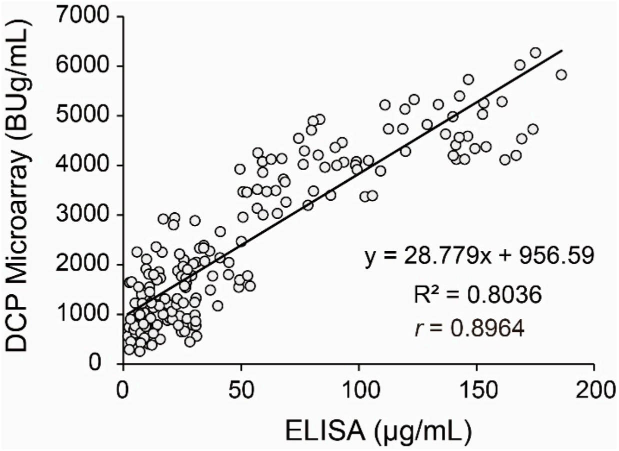

Figure S1. Correlation coefficients and equations of anti-SARS-CoV-2 IgG antibodies analyzed by ELISA (μg/mL) and DCP microarray (BUg/mL).

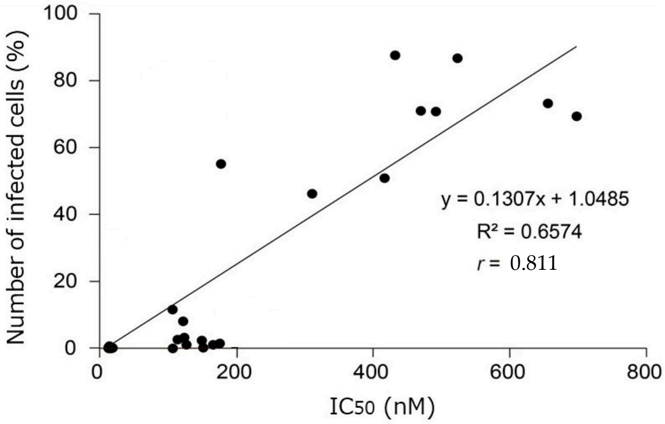

Figure S2. Correlation coefficient and equation of protective immunity analyzed by measurements of neutralizing activity of antibodies against pseudovirus and RBD binding avidity of anti-SARS-CoV-2 IgG antibodies.

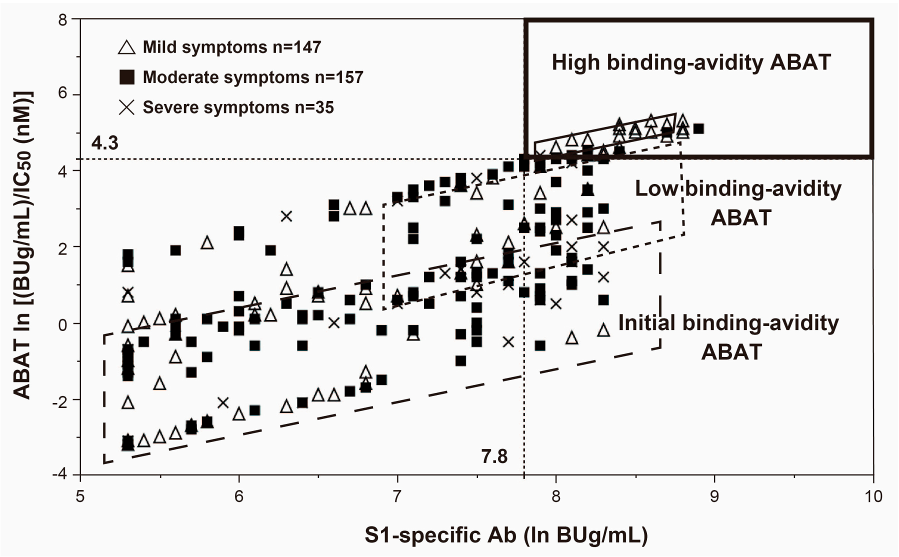

Figure S3. Assessment of the re-admission prevention potential of anti-SARS-CoV-2 IgG antibodies in patients after 2–3 weeks of hospitalization or at discharge from the hospital. Dotted lines show cutoff values of S1-specific antibody titer and ABAT for predicting prevention of hospitalization, as shown in Figure 6D. Ab, antibody.
